# From Our Correspondents

**Published:** 1988-02

**Authors:** 


### From Our Correspondents

**GERBIL Strikes at 3 Fundamental Academic Freedoms**

As I write, the universities are waiting with bated breath for the Committee stage discussions of the clauses of the Government's Education Bill (GERBIL) on February 9th. As you read this it is likely that the die will have been cast. If that means that the Bill is unamended then the future for higher education in this country is very bleak indeed.

This is potentially of great significance to readers of the journal. Sir Mark Richmond (i), chairman of the CVCP, has pointed out repeatedly that GERBIL strikes at the heart of three fundamental academic freedoms. These are the freedom to be protected from direct and narrow political interference by the government of the day; the freedom to research in subjects of as yet unrecognised importance; and thirdly, the freedom to question received wisdom. The Bill as it stands would abolish all three.

At the same time as this is happening the DHSS has introduced a clause into any research contract which it funds whereby no work deriving from that research can be published without the express permission of the secretary of State. The publication of a trial in which I was recently involved, has been held up because our collaborators are funded by the DHSS although the Department contributed no direct financial support for the project. Can you imagine the furore if a drug company tried to do the same?

Creeping centralisation and authoritarianism are totalitarian policies which must be opposed in principle and in practice. If you value your heritage of academic freedom you are going to have to fight for it.

1. RICHMOND, M (1988) What the Universities have done. *Nature*, 331, 403–4.

G. M. Stirrat

**'That's Life', Parliament and The Medical Royal Colleges: Organs for Transplantation**

Transplantation of kidneys and other organs has been one of the great advances of the last 25 years. For the failing kidney transplantation provides a better quality of life at lower cost than dialysis. For the failing heart, liver and lung, transplantation may be the only option.

There is a shortage of organs for transplantation. We think there are abut 4,000 brain stem deaths a year. About 2,500 kidneys are needed each year. The present transplant rate is around 1,800 a year. What can be done to increase the donor rate? As a member of Council of the Royal College of Physicians, I was asked to sit on a working party of the Medical Royal Colleges which met on six occasions in the early part of 1987, reported to the Department of Health and Social Security in June 1987 and whose report was published in December 1987. I had no direct involvement in renal failure and transplantation, no experience of brain stem death and no firm views when I joined the Committee about organ donation. I was made Secretary and had to learn fast!

I became convinced that brain stem death is death, that for most people brain stem death occurs when the heart stops beating and that for a very few the brain stem dies from a partially recovered cardiac arrest, from head injury, cerebral tumour or mengingitis. Such death is determined by clinical criteria; a patient in coma on a ventilator; a known diagnosis which can lead to brain stem death, the exclusion of confounding issues such as drugs, hypothermia and metabolic disorders and the confirmation by tests of brain stem function. I and we became convinced that there was no simple single solution to increasing organ donation. Good publicity helps, as shown by the 'That's Life' programme in 1985 on Ben Hardwick, following which transplants increased by 30%. Bad publicity on brain stem death hinders as shown by 'Panorama' in 1980 and the Sunday times in 1986, but fortunately bad publicity has a brief effect over several months: good publicity a sustained effect over several years. The special 'That's Life' programme in January 1988, has led to a record 100% increase in kidney donations and a considerable increase in other organ donations and this seems to be sustained. We need better education, more accessible information on suitability of donors and transplant arrangements, better coordination, particularly through a wider appointment of transplant coordinators, and greater facilitation of multiple organ donation by simplifying the surgical and theatre arrangements. Each hospital should establish procedures for organ donation and an audit of brain stem death, its outcome in organ donation and the reason for any shortfall. Above all, we need to improve communication with bereaved relatives. There is now a greater public awareness of the need for organs and over 80% of people have said that they agree to donation, though only 20% carry donor cards. There need be no fear of upsetting relatives by a sensitive approach which asks if the recently dead person would have wished their organs to be used to help others. We know that for many people such a gift is a consolation in their bereavement.

If all these things are done, there is no need for legally required request which in itself might not help for those who wish could evade the obligation and there could be no effective sanction. Yet a transplant notification bill was presented to Parliament and read for the first time on 3rd February 1985. A debate on transplant surgery took place on 26th February 1988. Those who want legislation believe that only a legally required request will achieve sufficient organ donations and that relatives have a right to be asked about organ donation. They cite American experiences over the last two years, but there is no certainty that the increase in organ donation is the result of the state laws and it is possible that it arises simply from increased publicity. Medicare and Medicaid have from October 1987, required participating hospitals to have a protocol for organ donation. The Working Party of the Medical Royal Colleges have recommended just that, the D.H.S.S. have accepted the recommendation. Watch for and encourage its implementation.

I. S. Bailey

**Two Cornish 'Firsts'**

A disgracefully long time ago your editor asked me to act as the Journal's Cornish correspondent. It seemed an easy enough task, to list the advantages and improvements in service which have been achieved in this most peripheral of districts. Since then the reality has been of plans postponed and projects shelved. Such gains as are being made are likely to be at the expense of the main structure of the service—a bit like building the battlements of a castle with stones taken from the foundations.

Most prominent of the battlements this year has been England's First Helicoptor Ambulance. To start with this was funded partly by sponsorship and partly by the District. The temptation to use the phrase "pilot trial" is overwhelming. District funding has now ceased and the future is uncertain, it will depend on whether the undoubted enthusiasm of the local community can be maintained and turned into hard cash. Its value in individual cases has been demonstrated and such a high profile venture has attracted the publicity accorded to a streaker at a Royal garden party but the cost is formidable. It costs as much to run a 40 hour a week service as it does to run a small cottage hospital.

Another 'first' and a source of local pride is the fact that Cornwall has been chosen as one of the guinea pig districts to run a mammography screening service. Rumour has it that there was strong competition but the fact that the clinicians concerned were able to present a previously agreed scheme (plus the feeling that if it can be made to work in Cornwall it will be a doddle elsewhere) seem to have won the day. The plan is for a mobile unit to visit all the larger communities in turn taking two or three years to complete the circuit of the district—those ignorant of geography should look at the map at this point. If the predictions of the Forrest report are accurate then, in the fullness of time the women of Cornwall will receive a 'Longevity Increment' of about fifty years. This previously unknown performance indicator is offered by your correspondent as a new year gift to our managers.

M. L. Cossfill

**The Work of Counsellors in General Practice**
**Occasional Paper 37**

The minimum amount of work in general practice associated with emotional disturbance is known to be about 7 per cent: some practitioners believe that it is as much as 30 per cent. While general practitioners need to be very sensitive to emotional problems, and can sometimes offer patients substantial help, they often have neither the time, the skills nor the training to do this.

As doctors have become more aware of the need to provide counselling, so counsellors have been emerging as professionals in their own right. Many are now attached to practices and this trend, taken with the decision of some family practitioner committees to accept counsellors under the ancillary staff reimbursement scheme and the White Paper's demand for general practice to accept a wider range of professions working within it, means that counselling in general practice is likely to become more common.

In a new *Occasional Paper, The Work of Counsellors in General Practice*, Dr June McLeod explores both the advantages and disadvantages of having a counsellor in general practice. She visited 14 practices and met 17 counsellors, and found that although there were some problems, there were also many gains for doctors, patients and counsellors.

*The Work of Counsellors in General, Occasional Paper 37* is available from the Central Sales Office, Royal College of General Practitioners, 14 Princess Gate, Hyde Park, London SW7 1PU, price £3.50 including postage. Cheques should be made payable to RCGP Enterprises Ltd.

Enquiries should be addressed to:
Dr June McLeod
17 Woodhill Road
Portishead
Bristol BS20 9EU

